# Characterisation of colistin resistance in Gram-negative microbiota of pregnant women and neonates in Nigeria

**DOI:** 10.1038/s41467-024-45673-6

**Published:** 2024-03-14

**Authors:** E. A. R. Portal, K. Sands, C. Farley, I. Boostrom, E. Jones, M. Barrell, M. J. Carvalho, R. Milton, K. Iregbu, F. Modibbo, S. Uwaezuoke, C. Akpulu, L. Audu, C. Edwin, A. H. Yusuf, A. Adeleye, A. S. Mukkadas, D. Maduekwe, S. Gambo, J. Sani, T. R. Walsh, O. B. Spiller

**Affiliations:** 1 Department of Medical Microbiology, Division of Infection and Immunity, Cardiff University, Cardiff, UK; 2 Ineos Oxford Institute for Antimicrobial Research, Department of Biology, University of Oxford, Oxford, UK; 3 Institute of Biomedicine, Department of Medical Sciences, University of Aveiro, Aveiro, Portugal; 4 Centre for Trials Research, Cardiff University, Cardiff, UK; 5 National Hospital Abuja, Abuja, Nigeria; 6 Murtala Muhammad Specialist Hospital, Kano, Nigeria; 7 Federal Medical Centre –Jabi, Abuja, Nigeria; 8 Interdisciplinary Biosciences DTP, University of Oxford, Oxford, UK; 9 Department of Medical Microbiology Aminu Kano Teaching Hospital, Kano, Nigeria; 10 Wuse General Hospital Abuja, Abuja, Nigeria; 11 Department of Paediatrics, Murtala Muhammed Specialist Hospital, Kano, Nigeria; 12 Department of Paediatrics Abdullahi Wase Teaching Hospital, Kano, Nigeria

**Keywords:** Antimicrobial resistance, Bacterial genes

## Abstract

A mobile colistin resistance gene *mcr* was first reported in 2016 in China and has since been found with increasing prevalence across South-East Asia. Here we survey the presence of *mcr* genes in 4907 rectal swabs from mothers and neonates from three hospital sites across Nigeria; a country with limited availability or history of colistin use clinically. Forty mother and seven neonatal swabs carried *mcr* genes in a range of bacterial species: 46 *Enterobacter* spp. and single isolates of; *Shigella*, *E. coli* and *Klebsiella quasipneumoniae*. Ninety percent of the genes were *mcr-10* (*n* = 45) we also found *mcr-1* (*n* = 3) and *mcr*-*9* (*n* = 1). While the prevalence during this collection (2015-2016) was low, the widespread diversity of *mcr*-gene type and range of bacterial species in this sentinel population sampling is concerning. It suggests that agricultural colistin use was likely encouraging sustainment of *mcr*-positive isolates in the community and implementation of medical colistin use will rapidly select and expand resistant isolates.

## Abstract (web summary)

Here, the authors report the results of a BARNARDS sub-study identifying a 1% mobile colistin resistance gene (mcr) carriage rate in around 5000 rectal swabs from mothers and neonates across Nigeria, of which 90% were mcr-10 (mostly *Enterobacter* spp.) and 10% were mcr-1 and mcr9.

## Introduction

Colistin, a polymyxin antibiotic rarely used clinically until recently due to associated nephrotoxicity, is now prescribed more often due to increasing global prevalence of multidrug resistant (MDR) infections lacking alternative antibiotic options^1,2^. The mobile colistin resistance (*mcr*) gene was initially identified as the underlying cause of colistin resistance in *Escherichia coli* isolated from an intensive pig farm in Shanghai, China^3^. It was subsequently linked to the use of colistin in intensive farming as a growth promotor, and its discovery led to the colistin agricultural ban in November 2016^4^. Unfortunately, subsequent surveillance suggests colistin use in farming remains high in South-East Asia^5–7^ and a recent report suggests colistin may be used in agriculture in some African countries^8^.

A review article by Anyanwu et al. published in 2021 summarises *mcr* prevalence in different ecosystems in Africa^9^ and found that unregulated use of antibiotics (including colistin) was directly linked to environmental and animal sources where bacteria carrying *mcr* genes had been identified. *mcr* positive *E. coli* have been detected in human, chicken, and poultry environments^3^, and subsequent publications have found transfer between *E. coli* strains and other species on a variety of plasmid backgrounds (including IncHI2, IncI2, IncX4, and IncP) via aquaculture, supply chains and slaughterhouses^10^. To date, 10 *mcr* genes (*mcr-1* to *mcr-10*) with minor variants have been characterised^11^, and *mcr* has been reported globally^12,13^.

Widescale surveillance of colistin resistance became relatively common following the discovery of *mcr*, with a predominance of monitoring in wealthy regions across North America and Europe^14–20^, aided by the wealth of the health services. However, there is a dearth of information regarding the prevalence and spread of *mcr* in Africa.

*mcr* was first described in Africa in 2016 in a South African study evaluating the presence of *mcr-1* in broiler chickens^21^. A literature review on PubMed (gated between 2014–2023) searching for “colistin resistance + Africa” yielded 298 results, reduced to 164 hits when “*mcr* + Africa was searched”. A literature review on PubMed searching for “*mcr*/colistin” + “Nigeria” yielded 29 hits, of which three collectively contained microbiology data^19,22,23^ suggesting that screening for colistin resistance, particularly from human carriage and infection is scarce. Whereas, an investigation in Southern Nigeria showed a prevalence of *mcr-1* in poultry of 2.9%^19^.

BARNARDS is a study analysing neonatal sepsis comprising of a network of 12 clinical sites in seven low- and middle-income countries (LMICs)^24–27^. Mothers and their respective neonates were enrolled into the BARNARDS study between November 2015-December 2017. In addition to examining bacterial causes and risk factors of neonatal sepsis, within BARNARDS the maternal and neonatal rectal microbiota were screened for the presence of extended spectrum ß-lactamases (ESBL) and carbapenemase antibiotic resistance genes (ARG)^28^. Previous studies have shown that the carriage of AMR gram-negative bacteria (GNB) is not only increasing but is both a risk factor and a precursor to infection, particularly in hospitalised or immunocompromised patients^29–31^. Although our previous work in BARNARDS suggests colistin wasn’t used clinically in Nigeria during the study period, it is possible colistin was imported for use in agriculture and farming^9,32^.

The aim of this sub-study was firstly to retrospectively screen maternal and neonatal swabs from the three clinical sites in Nigeria (two in Abuja, and one in Kano) for the presence of *mcr*, and secondly to phenotypically and genotypically characterise bacterial isolates harbouring *mcr* gene variants.

## Results

### Mother/neonate characteristics and *mcr* carriage

**Fig. 1:**
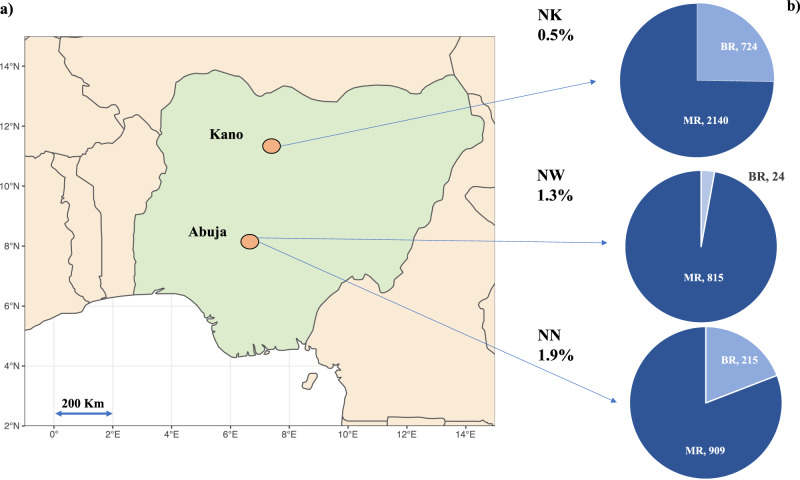
An infographic map indicating the location of sampling, number of samples per type, and prevalence of *mcr*. **a** Shows a map of Nigeria highlighting the location of the three hospital sites NK Kano Nigeria: NN: National Hospital Abuja, Nigeria, NW: WUSE hospital Abuja, Nigeria, with (**b**) embedded pie charts showing the prevalence of *mcr* and the split proportion and number of which were maternal rectal samples (MR) and neonatal rectal swabs (BR). NN and NW are both in Abuja and are 8 km apart, NN to NK is 350 km. The map of Nigeria was created using ggplot2 and map_data in R.

In total, 4907 rectal swabs (*n* = 3944 Mother Rectal [MR] and *n* = 963 Baby Rectal [BR]) were processed from hospitals in Kano (NK) and Abuja (NN or NW) (NK-MR *n* = 2140, NK-BR *n* = 724, NN-MR *n* = 909, NN-BR *n* = 215, NW-MR *n* = 895, NW-BR *n* = 24) (Fig. 1) and microbial growth was detected in 70% (*n* = 3,435) following swab enrichment for 48 h in vancomycin and colistin broth. Following screening carried out by PCR to detect *mcr-1, mcr-3, mcr-8-10*, 1% of samples (*n* = 46) were positive for at least one *mcr* gene (Fig. 1b). Forty-nine distinct bacterial isolates (41 MR and 8 BR) were recovered between 18th November 2015 and the 24th October 2017 (Table 1). The prevalence of *mcr* carriage was similar between MR and BR, at 1% (*n* = 39/3944) and 0.7% (*n* = 7/963) respectively (Fig. 1b). Forty mothers and seven neonates had *mcr* within their gut microbiome, however we did not find a case whereby both the mother and corresponding neonate both carried *mcr*. Of the three hospitals where mothers’ and neonates’ rectal microbiota were screened, NN had the highest prevalence (*n* = 21/1124, 1.9%), with *mcr* carriage in samples collected from NW at similar rates (*n* = 12/919, 1.3%) (Fig. 1b), compared to a lower incidence in NK (*n* = 13/2864, 0.5%).

**Table 1:** A summary of samples that were positive for an *mcr* antibiotic resistance gene (ARG), listing the hospital site and sample type, date, and clinical epidemiological data including antibiotic usage

Isolate code^a^	ENA Accession^b^	ABX^c^	Type of living^d^	Outcome^e^	Neonatal sepsis case^f^
NK-BR1052-1	ERS14349763	AMX, GEN	Semi rural	Alive	Y
NK-BR1540	ERS14349816	ND	Urban	Alive	N
NK-BR1684A	ERS14349742	ND	Urban	Alive	N
NK-BR6054-1	ERS14349781	ND	Urban	Alive	N
NK-BR6054-2	ERS14349782	ND	Urban	Alive	N
NK-MR1571	ERS14349826	NA	Urban	Untraceable	Y
NK-MR1610	ERS14349747	NA	Urban	Alive	Y
NK-MR2027	ERS14349827	NA	Urban	Untraceable	N
NK-MR2917	ERS14349865	NA	Semi rural	Alive	N
NK-MR4285	ERS14349828	NA	Urban	Alive	N
NK-MR4949	ERS14349829	NA	Urban	Alive	N
NK-MR5457	ERS14349830	NA	Urban	Alive	N
NK-MR5472	ERS14349785	NA	Semi rural	Alive	N
NK-MR5868	ERS14349831	NA	Semi rural	Alive	N
NN-BR146	ERS14349788	AMX, CEF	Urban	Alive	Y
NN-BR158	ERS14349750	AMX, CEF	Urban	Alive	Y
NN-BR1663r1	ERS14349790	ND	Semi rural	Alive	N
NN-MR1252	ERS14349797	NA	Urban	Alive	N
NN-MR1335	ERS14349753	NA	Urban	Alive	Y
NN-MR149	ERS14349839	NA	Urban	Alive	N
NN-MR1507	ERS14349840	NA	Urban	Deceased	N
NN-MR1686	ERS14349800	NA	Urban	Alive	N
NN-MR1778	ERS14349801	NA	Urban	Alive	N
NN-MR196	ERS14349841	NA	Urban	Alive	Y
NN-MR291	ERS14349803	NA	Urban	Alive	N
NN-MR4	ERS14349843	NA	Urban	Alive	N
NN-MR49-B	ERS14349873	NA	Urban	Deceased	N
NN-MR49-P	ERS14349870	NA	Urban	Deceased	N
NN-MR673	ERS14349846	NA	Urban	Alive	N
NN-MR759	ERS14349847	NA	Semi rural	Alive	N
NN-MR765	ERS14349848	NA	Urban	Alive	N
NN-MR770-1	ERS14349849	NA	Urban	Alive	N
NN-MR770-2	ERS14349850	NA	Urban	Alive	N
NN-MR773	ERS14349851	NA	Urban	Alive	N
NN-MR781	ERS14349852	NA	Urban	Alive	N
NN-MR803	ERS14349806	NA	Urban	Alive	N
NN-MR840	ERS14349853	NA	Urban	Alive	N
NW-MR118	ERS14349854	NA	Urban	Alive	N
NW-MR1265	ERS14349804	NA	Rural	Alive	N
NW-MR1280	ERS14349855	NA	Urban	Alive	N
NW-MR132	ERS14349857	NA	Urban	Deceased	N
NW-MR1409	ERS14349805	NA	Urban	Alive	N
NW-MR1424	ERS14349858	NA	Rural	Alive	N
NW-MR1609	ERS14349876	NA	Rural	Alive	N
NW-MR1805	ERS14349869	NA	Urban	Alive	N
NW-MR554	ERS14349861	NA	Urban	Alive	N
NW-MR576	ERS14349862	NA	Urban	Alive	N
NW-MR858	ERS14349755	NA	Urban	Alive	N
NW-MR986	ERS14349863	NA	Urban	Alive	N

No colistin, aminoglycosides or fluoroquinolones, were reported used in the three months prior to birth.

^a^Isolate code.

^b^ENA accession.

^c^Neonate antibiotics.

^d^Mother reported type of living.

^e^Neonatal outcome up to 60 days.

^f^Denotes whether rectal sample was linked to neonatal sepsis.

*NN* National hospital, *NW* Wuse Hospital, *NK* Kano, *MR/BR* mother/neonate, *ND* no data, *NA* not applicable, *AMX* amoxicillin, *GEN* gentamicin, *CEF* ceftazidime.

In total, 32/39 mothers who carried *mcr* reported living in an urban environment (compared to rural, *n* = 3 and semi-rural, *n* = 4). Seven mothers reported previous antibiotic use including ß-lactams, macrolides, and metronidazole. Three of the seven neonatal sepsis cases (Table 1) were reported to have blood culture confirmation, and none of the *Enterobacter* reported from rectal carriage in this study were the causative sepsis pathogen. The three neonates with neonatal sepsis were empirically treated with either amoxicillin and ceftazidime (*n* = 2/3) or amoxicillin and gentamicin, (*n* = 1/3). Colistin was not prescribed to any mother or neonate (from available data) within this study.

### Diversity of colistin resistant bacterial isolates

**Fig. 2:**
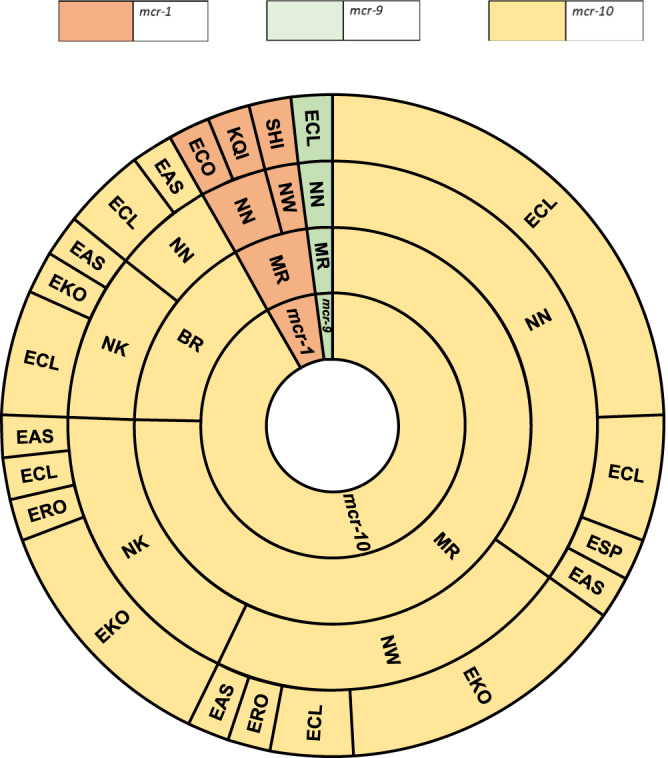
Bacterial species distribution per hospital site, sample type and *mcr* variant. A sunburst diagram delineating the *mcr* gene in the context of the sample type (MR – mother rectal, BR – baby rectal), hospital site (National Hospital Abuja NN, Wuse District Hospital Abuja NW, Murtala Mohammad Specialist Hospital Kano NK) and bacterial species. EAS *Enterobacter asburiae*, ECL *Enterobacter cloacae*, EKO *Enterobacter kobei*, ERO *Enterobacter roggenkampii*, ESP *Enterobacter* sp., ECO *Escherichia coli*, KQI *Klebsiella quasipneumoniae*, SHI *Shigella* sp. Forty-nine bacterial isolates carrying an *mcr* gene are represented in this sunburst diagram.

Of the 70% (*n* = 3435) of swabs producing microbial growth, 133 bacterial isolates were cultured from the colistin selective media (4 mg/L) screening. Forty-nine isolates carried an *mcr* gene, and short-read WGS was completed for all (Table 1, Fig. 2) with complementary long-reads generated for 16 isolates. We detected five *Enterobacter* spp. making up 94% (*n* = 46/49) of the *mcr* positive cohort. Twenty-six were *Enterobacter kobei*, 11 *Enterobacter cloacae*, five *Enterobacter asburiae*, two *Enterobacter roggenkampii* and two *Enterobacter* spp. The remaining three isolates were *Shigella* (*n* = 1), *E. coli* (*n* = 1) and *K. quasipneumoniae* (*n* = 1) (Fig. 2). *mcr-10* was the most frequently detected gene variants and accounted for 92% (*n* = 45/49) of all rectal samples positive for *mcr* (Fig. 2). The remaining *mcr* genes detected were *mcr-1* (*n* = 3, 6%), and *mcr-9* (*n* = 1, 2%). *mcr-8* was not detected in any of the samples, and although we detected a PCR positive hit for *mcr-3*, following WGS analysis this was not confirmed as *mcr-3* (Supplementary Fig. 1). Eight isolates with *mcr* genes were recovered from seven neonates, all were *Enterobacter* species, and all carried *mcr-10*.

There were 17 different STs detected within the *mcr* positive *Enterobacter* isolates, with the most common being ST1, ST125 and ST691. *mcr* positive *E. cloacae* ST1 were recovered from mother and baby rectal swabs from all three Nigerian sites, but the majority were from mothers enrolled into hospitals in Abuja. Whereas *E. kobei* ST691 with *mcr-10* were largely recovered from mothers enrolled into Kano, *E. kobei* ST125 with *mcr-10* were all isolated from mothers enrolled in a single hospital site in Abuja (NN-MR), and all except one, were cultured from samples collected in August 2016 (*n* = 6).

From the *mcr* negative and colistin resistant cohort, we identified *n* = 80/83 *Enterobacter* species, (*n* = 32 *E. cloacae*, *n* = 22 *E. kobei*, *n* = 10 *E. asburiae* and *n* = 16 other *Enterobacter* species) and a single isolate of each *Shigella sonnei*, *K. quasipneumoniae* and *Raoultella ornithinolytica*. During WGS analysis there were a total of 43 previously undetected STs classified; ST1601-ST1608 (*mcr* positive) and ST1864-1895 (*mcr* negative).

### Antimicrobial resistance

**Fig. 3:**
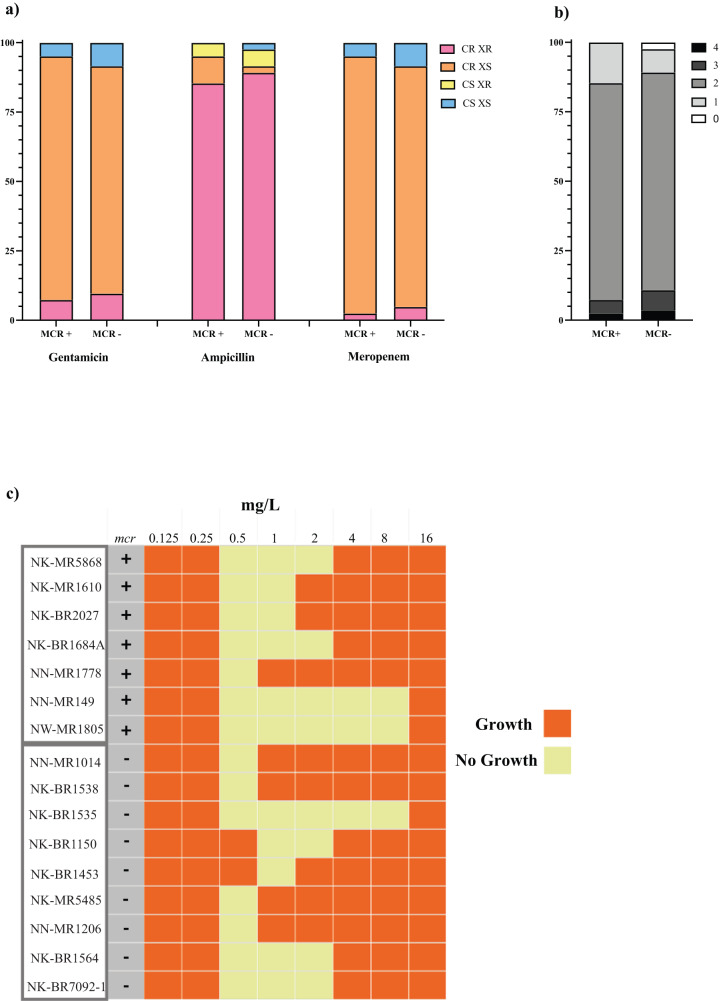
Antimicrobial resistance of bacterial isolates purified from mother and neonatal samples. **a** Stacked bar graph showing the MIC profile percentages of *mcr* positive isolates (*n* = 41) and *mcr* negative isolates (*n* = 83) screened against colistin, ampicillin, gentamicin and meropenem. Blue denotes isolates sensitive to both colistin and ampicillin, gentamicin or meropenem CS XS. Yellow denotes isolates sensitive to colistin and resistant to ampicillin, gentamicin or meropenem CS XR. Orange denotes isolates resistant to colistin and sensitive to either ampicillin, gentamicin or meropenem CR XS. Pink denotes isolates resistant to both colistin and ampicillin, gentamicin or meropenem CR XR. **b** A stacked bar graph showing the percentage profile of isolates that were resistant to how many 0–4 of the antibiotics tested colistin and ampicillin, gentamicin or meropenem. Split by *mcr* positive isolates (*n* = 41) and *mcr* negative isolates (*n* = 83). 0 (white) shows no resistance and 4 (black) shows resistance to all 4 tested antibiotics. **c** A heatmap highlighting the skipped well phenomena^33^ in *Enterobacter* spp., showing that the bacteria cease growing at low concentrations (no growth depicted as yellow) of colistin but later return at higher concentrations (depicted in orange), this also indicates this is not an effect restricted to *mcr* positive isolates as this was observed in both *mcr* positive and *mcr* negative isolates.

Minimum inhibitory concentrations (MICs) were determined for 41 *mcr* positive and 83 *mcr* negative isolates (*n* = 124 total) as shown in Fig. 3a, Supplementary Data 1. In addition to determining the MIC for colistin, three additional antibiotics were also selected for antimicrobial susceptibility testing: ampicillin, gentamicin and meropenem. Carbapenem resistance was reported during characterisation of clinical bacterial isolates collected during BARNARDS^27^ and, ampicillin and gentamicin are the empirical first line treatment options for neonatal sepsis as per the WHO guidelines. We expected colistin resistance to be high due to enrichment with colistin, and report this at 93% (*n* = 115/124). Likewise, ampicillin resistance was high at 94% (*n* = 116/124) but this was also unsurprising as most of these (95%) (*n* = 118/124) were intrinsically resistant *Enterobacter*. All *Enterobacter cloacae* contained *bla*_CMH-3_ and *Enterobacter asburiae*, *hormaechei* and *kobei* isolates all contained *bla*_ACT-2_ *bla*_ACT-4_, *bla*_ACT-6_, or *bla*_ACT-9_. Of the non-*Enterobacter* species, *n* = 5/6 were resistant to ampicillin and all six isolates carried at least one β-lactamase gene. Eleven isolates were resistant to gentamicin, ampicillin and colistin (*n* = 8/11 were *mcr* negative). Of these *n* = 4/11 were also resistant to meropenem, however only one isolate contained a carbapenemase ARG *bla*_NDM-5_. NW-MR576 for example carried multiple ARGs within the same plasmid including *bla*_TEM-1_, *aph-(3”)-lb*, *tet*(A) and *catA1*, ultimately conferring resistance to several different antibiotic classes. The majority of isolates tested (*n* = 97/124) were resistant to *n* = 2/4 antibiotics tested (Fig. 3b), with 8 isolates resistant to *n* = 3/4 antibiotics and 4 isolates were resistant to all antibiotics tested (Fig. 3b).

*mcr-1.1* conferred colistin resistance in all three isolates, two of which were from a single rectal swab (NN-MR49-1 (*E. coli*) and NN-MR49-2 (*K. quasipneumoniae*)). These isolates also contained several aminoglycoside, sulphonamide, and tetracycline ARGs (Table 2), many of which were located on the same plasmid as *mcr-1.1*. These two *mcr-1.1* containing isolates were resistant to gentamicin and ampicillin but sensitive to meropenem. A *Shigella* spp. (NW-MR1609) containing *mcr-1.1* in addition to chromosomally located *bla*_CTX-M-15_ and *qnrS1*, (Table 2) was sensitive to gentamicin and meropenem but resistant to ampicillin.

**Table 2:** A summary of bacterial isolates containing *mcr* antibiotic resistance genes (ARG), including the sample type, hospital location, date, bacterial species, sequence type (ST), and ARG profile

*mcr* variant	Isolate	Species	MLST	Hybrid genome accession (NCBI)	Inc type^a^	size bp^b^
*mcr-1.1*	NN-MR49-1	*Escherichia coli*	1266	JAWPEQ000000000	IncHI2, TrfA	293,168
*mcr-1.1*	NN-MR49-2	*Klebsiella quasipneumoniae*	NA	JAWPEP000000000	IncHI2, TrfA	294,497
*mcr-1.1*	NW-MR1609	*Shigella sp*.	484	JASARV000000000	IncX4	33,281
*mcr-10*	*NN-BR158	*Enterobacter asburiae*	162	JANFVO000000000	IncFIB, IncN	191,776
*mcr-10*	NW-MR858	*Enterobacter asburiae*	339	N/A	N/A	N/A
*mcr-10*	*NK-BR1684A	*Enterobacter asburiae*	1602	JANFVD000000000	IncFII, IncFIA	162,389
*mcr-10*	NN-MR1335	*Enterobacter asburiae*	1606	JANUGO000000000	N/A	N/A
*mcr-10*	NK-MR1610	*Enterobacter asburiae*	1607	JANFVN000000000	DNA fragment	15,560
*mcr-10*	*NK-BR1052-1	*Enterobacter cloacae*	1	JANFVM000000000	IncR	113,734
*mcr-10*	NN-MR1252	*Enterobacter cloacae*	1	N/A	N/A	N/A
*mcr-10*	NW-MR1265	*Enterobacter cloacae*	1	N/A	N/A	N/A
*mcr-10*	NW-MR1409	*Enterobacter cloacae*	1	N/A	N/A	N/A
*mcr-10*	NN-MR1686	*Enterobacter cloacae*	1	N/A	N/A	N/A
*mcr-10*	NN-MR291	*Enterobacter cloacae*	1	N/A	N/A	N/A
*mcr-10*	NN-BR146	*Enterobacter cloacae*	84	N/A	N/A	N/A
*mcr-10*	NK-BR6054-1	*Enterobacter cloacae*	84	N/A	N/A	N/A
*mcr-10*	*NK-BR6054-2	*Enterobacter cloacae*	84	JANFVL000000000	IncFIB	118,078
*mcr-10*	NK-MR5472	*Enterobacter cloacae*	84	N/A	N/A	N/A
*mcr-10*	NN-MR1778	*Enterobacter sp.*	1604	N/A	N/A	N/A
*mcr-10*	NN-MR673	*Enterobacter kobei*	32	N/A	N/A	N/A
*mcr-10*	*NK-BR1540	*Enterobacter kobei*	56	JANFVK000000000	IncFIB	65,562
*mcr-10*	*NK-MR5457	*Enterobacter kobei*	56	JANFVJ000000000	IncFIB, IncR	101,412
*mcr-10*	NN-MR4	*Enterobacter kobei*	56	N/A	N/A	N/A
*mcr-10*	NN-MR840	*Enterobacter kobei*	56	N/A	N/A	N/A
*mcr-10*	NW-MR1424	*Enterobacter kobei*	56	N/A	N/A	N/A
*mcr-10*	NW-MR986	*Enterobacter kobei*	56	N/A	N/A	N/A
*mcr-10*	NK-MR2027	*Enterobacter kobei*	56	N/A	N/A	N/A
*mcr-10*	NN-MR773	*Enterobacter kobei*	125	JANFVI000000000	IncFIB, IncFII, IncR	121,458
*mcr-10*	NN-MR759	*Enterobacter kobei*	125	N/A	N/A	N/A
*mcr-10*	NN-MR765	*Enterobacter kobei*	125	N/A	N/A	N/A
*mcr-10*	NN-MR770-1	*Enterobacter kobei*	125	N/A	N/A	N/A
*mcr-10*	NN-MR770-2	*Enterobacter kobei*	125	N/A	N/A	N/A
*mcr-10*	NN-MR781	*Enterobacter kobei*	125	N/A	N/A	N/A
*mcr-10*	NN-MR196	*Enterobacter kobei*	125	N/A	N/A	N/A
*mcr-10*	NK-MR5868	*Enterobacter kobei*	691	N/A	N/A	N/A
*mcr-10*	NK-MR4949	*Enterobacter kobei*	691	N/A	N/A	N/A
*mcr-10*	NN-MR1507	*Enterobacter kobei*	691	N/A	N/A	N/A
*mcr-10*	NW-MR118	*Enterobacter kobei*	691	N/A	N/A	N/A
*mcr-10*	NW-MR554	*Enterobacter kobei*	691	N/A	N/A	N/A
*mcr-10*	NK-MR4285	*Enterobacter kobei*	691	N/A	N/A	N/A
*mcr-10*	NK-MR1571	*Enterobacter kobei*	691	N/A	N/A	N/A
*mcr-10*	NN-MR149	*Enterobacter kobei*	691	N/A	N/A	N/A
*mcr-10*	NW-MR132	*Enterobacter kobei*	1601	JANFVH000000000	N/A	N/A
*mcr-10*	*NW-MR576	*Enterobacter kobei*	1605	JANFVG000000000	IncFIB	139,705
*mcr-10*	NW-MR1280	*Enterobacter kobei*	ND	N/A	N/A	N/A
*mcr-10*	NK-MR2917	*Enterobacter roggenkampii*	1237	JANFVF000000000	N/A	N/A
*mcr-10*	NW-MR1805	*Enterobacter roggenkampii*	1237	N/A	N/A	N/A
*mcr-9*	NN-MR803	*Enterobacter sp.*	1603	JAWPER000000000	IncFII, IncFIB	186,925

*Denotes isolates that were used for the conjugation experiment.

^a^Denotes the plasmid replicon types detected in the mcr containing plasmid.

^b^Denotes the size (in bp) of the mcr containing plasmid.

*N/A* Not applicable, *ND* Data was not available.

A well reported “skipped well” phenomena^33^ was observed in colistin MIC testing where the growth of *Enterobacter* was inhibited at certain concentrations, however growth was visible at higher MIC concentrations (Fig. 3c). These were exclusively observed in *Enterobacter* and were apparent in both *mcr* positive and *mcr* negative isolates (Fig. 3c). The single *mcr-9 Enterobacter* isolate remained sensitive to colistin (MIC = 0.5 mg/L), gentamicin and meropenem, the only other ARG detected were intrinsic, including *fosA* and *bla*_ACT-6_. All *Enterobacter* isolates containing *mcr-10*, bar one, were resistant to colistin. One (NK-BR1684) of these isolates was also resistant to gentamicin and meropenem, however it did not contain any known resistance genes to aminoglycosides or carbapenems.

### mc*r-1* genetic context

**Fig. 4:**
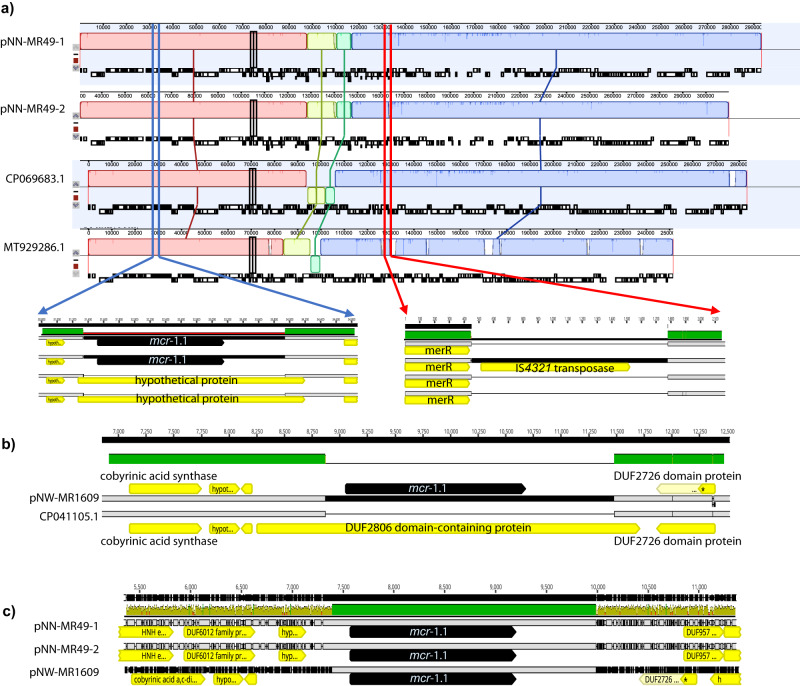
Genetic context of *mcr-1*-positive bacterial isolates. **a** A comparative analysis using Mauve of two *mcr-1.1* containing IncHI2A plasmids from the same sample; *E. coli* (pNN-MR49-1, 293,168 bp) ST1266, and a *K. quasipneumonaie* (pNN-MR49-2, 294,497 bp) compared to two similar non-*mcr* carrying plasmids accession number NZ_CP069683.1 (from Poland) and NZ_MT929286.1 (from Czech Republic). **b** The genetic context of the *mcr-1.1 Shigella* spp. (pNW-MR1609) ST484 IncX4 plasmid relative to *Klebsiella pneumoniae* IncX4 plasmid (accession number CP041105) from Thailand, where the *mcr*−1.1 gene was the only major difference. **c** Despite the very different plasmid backbones for our 3 *mcr*-containing plasmids they all share an identical 2602 bp without evidence of IS element or transposase presence.

There were two MR samples positive for *mcr-1.1* with three bacterial isolates recovered (Table 1). From sample NN-MR49 both an *E. coli* (NN-MR49-1) ST1266, and a *K. quasipneumoniae* (NN-MR49-2) isolate, both carrying *mcr-1.1* on large IncHI2A plasmids of 293,168 bp (*E. coli)* and 294,497 bp (*K. quasipneumoniae*) were cultured (Table 2). Comparative analysis of plasmid assemblies using Mauve alignment revealed a large degree of similarity between the plasmids (Fig. 4a). Following Blastn and PLSDB comparison, NZ_CP069683.1 (Poland) and NZ_MT929286.1 (Czech Republic) were found to be the most similar.

NZ_CP069683.1 and the slightly smaller NZ_MT929286.1 plasmids were very similar except that they did not contain *mcr-1.1* but appear as a 2602 bp insert in an identical hypothetical protein relative to both the *mcr*-negative plasmids. Isolates from NN-MR-49 carried near-identical *mcr-1.1* plasmids in *E. coli* and *K. quasipneumoniae*, differing only by 19 SNPs and one 1335 bp insertion between pNN-MR49-1 and pNN-MR49-2. The latter took the form of an IS*4321* element in the *K. quasipneumoniae* isolate (Fig. 4a) between the mer operon (merR) for mercury resistance and the tetracycline repressor protein (*TetR*) of the adjacent *tet(A)* gene. The IS*4321* element was not found in any of the *E. coli* sequences (JAWPEQ00000000) but was present in three separate plasmids carried by the *K. quasipneumoniae* sequence (JAWPEP000000000; including the *mcr-1.1* carrying plasmid) suggesting that the IS*4321* transposase had been acquired from one of these other two plasmids.

Sample NW-MR1609 contained a *Shigella* ST484 carrying *mcr-1.1* on a 33,281 bp IncX4 plasmid (pNW-MR1609), which was highly homologous to a 31,229 bp IncX4 plasmid isolated from a swine rectal swab in Thailand (accession number CP041105) that lacked the *mcr-1.1* gene (Fig. 4b). Despite being found in three completely different species and two different Inc plasmid backgrounds the *mcr*−*1.1* gene was found on an identical 2602 bp segment without any evidence of an associated IS element or transposase (Fig. 4c). Highly homologous plasmids lacking the *mcr-1.1* gene were found for these plasmids and no homology or insert boundary repeat sequences (direct or indirect) could be found for the genes that were disrupted at the insertion site (Fig. 4a, b). Following Geneious (v2022.1.1) and Blastn analysis (v2.7.1) we found a similarity between pNW-MR1609 and IncX4 plasmid sequences from *mcr-1.1* positive *E. coli* and *K. pneumoniae* from a Nigerian study conducted by Ngbede et al.^23^. Ngbede performed WGS on 15 isolates, and assembly data produced contigs ~30 Kb allowing comparison of the plasmid backbone. Furthermore, following Blastn analysis, we detected >100 hits with greater than 99 % coverage and nucleotide identity suggesting the 33 Kb IncX4 *mcr-1.1* containing plasmid has been well disseminated. Analysis within PLSDB reveals that similar IncX4 plasmids have been isolated from various sectors including animals and humans from Denmark, China, Egypt, USA, and Vietnam, likewise reported in the literature^34–37^.

### *mcr-9* genetic context

**Fig. 5:**
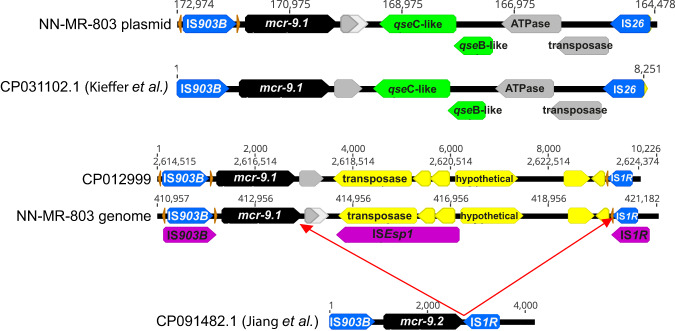
Two copies of *mcr-9* (black arrows) were found in isolate NN-MR-803, one on a plasmid and one inserted into the chromosome. A previous publication by Tyson et al.^39^, had defined three separate cassettes defined by IS elements IS903B and IS*1R* (blue arrows). The *mcr-9* gene carried on the plasmid was completely homologous to the “type A” configuration in their Fig. 3, including the qseB-like and qseC-like genes (green arrows) required for induction of colistin resistance. However, while the NN-MR-803 genomic copy of *mcr-9* had similarity to Tyson et al.’s^39^ Fig. 3C, an additional intervening ISEsp1 insertion was observed (ISfinder identified genes shown as purple arrows). However, an identical *mcr-9* configuration was found on the chromosome of GenBank accession number CP012999 *Enterobacter* sp. Isolate E20. Conserved cupin fold metalloprotein, WbuC is shown as a grey arrow and other open reading frames are shown as yellow arrows (including a transposase overlapping ISEsp1 from GenBank annotation). Large discrepancies between genomic and plasmid environments surrounding *mcr-9* indicates independent acquisition of these from separate sources.

Two identical copies of *mcr-9.1* were detected in NW-MR803 an *Enterobacter* isolate most closely related to *E. cloacae*. One was found on the chromosome and one on a 186,925 bp IncFII/IncFIB plasmid (Fig. 5). Kieffer et al.^38^, showed that *mcr-9.1* expression is inducible (with associated elevated colistin MIC) in the presence of colistin when *mcr-9* is located upstream of the *qseBC* two-component regulatory system (present upstream of our plasmid *mcr-9.1*), but Tyson et al.^39^, disputed the capacity of *qseBC* to mediate upregulation. Only the plasmid borne *mcr-9* had these adjacent genes, present on an 8251 bp mobile element flanked by IS elements IS*903B* and IS*26* (Fig. 5). However, on the chromosome, the *mcr-9.1* gene is located upstream from an IS*1R* locus (Fig. 5), which Jiang et al.^40^, have recently shown could mediate upregulation of *mcr-9.2* in Genbank accession CP091482.1. Although the genomic cassette containing *mcr-9.1* in NW-MR803 additionally appears to have been further modified with the adjacent insertion of an IS*Esp1* element (which is also present in GenBank accession CP012999 *Enterobacter* sp. Isolate E20) indicating independent acquisition of these *mcr-9.1* genes between plasmid and genome. Colistin MIC was found to be 2 mg/L as determined by broth microdilution, which was at the EUCAST breakpoint threshold (resistance >2 mg/L). However, a single passage in 0.5 mg/L colistin resulted in elevation of the MIC to 8 mg/L and a second passage elevated colistin MIC of 32 mg/L (Supplementary Fig. 2). However, no further elevation of MIC was observed with subsequent passages in sub-MIC colistin.

### *Mcr-10* genetic context

**Fig. 6:**
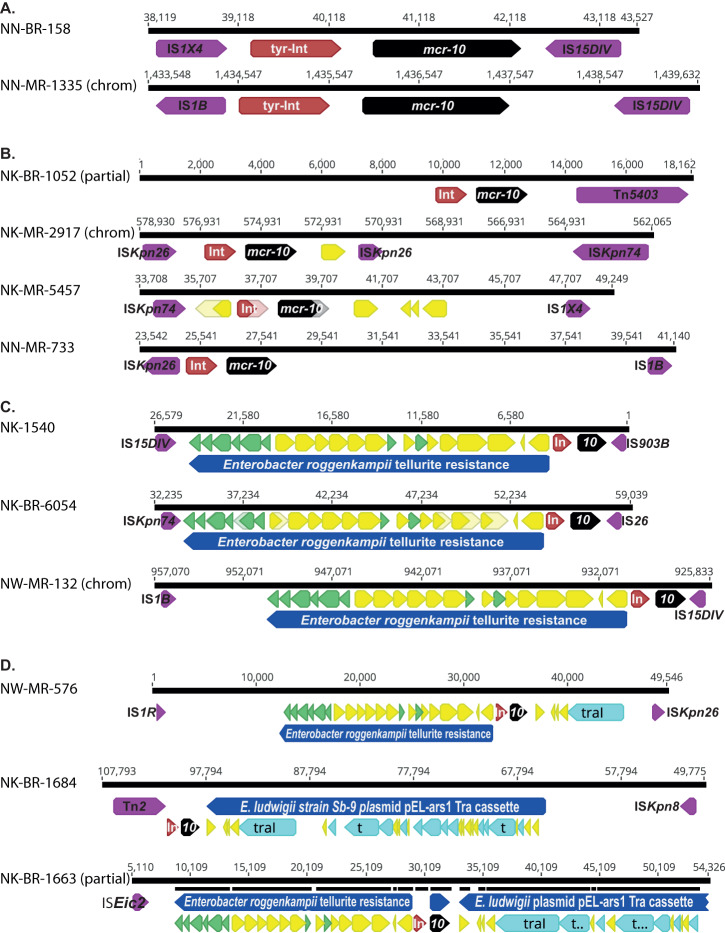
Twelve isolates were found to carry *mcr-10* (black arrows), nine on plasmids and three on chromosomal (chrom) insertions. In all occurrences the resistance gene was confined to a conserved 3378 bp region that included an adjacent tyrosine-type integrase (tyr-Int or In; brown arrows), defining the smallest unit transposon. Analysing the adjacent environment using ISfinder these unit transposons were located on inserts of various lengths bordered by a variety of IS elements (purple arrows). These could largely be classified as (**A**) minimal: 5132–5902 bp; **B** small: 14,539-17,400 bp (although NK-BR1052 was truncated by contig assembly); **C** intermediate: 25,862–30,548 bp (which includes a region of 20,215 bp homologous) to a *Enterobacter roggenkampii* plasmid (GenBank Accession number CP116250) containing a cassette of tellurite resistance genes (green arrows); and (**D**) complex regions of >50,000 bp, two of which include the *Enterobacter roggenkampii* plasmid tellurite resistance cassette, as well as genes from the *tra* family of conjugal elements (light blue arrows). Two of these isolates have >20,000 bp regions with high homology to a *tra* gene family cassette carried by an unnamed plasmid in *Enterobacter* sp. Z1 (Genbank accession number CP099720). No consistent pattern of boundary IS elements could be discerned to account for a larger conserved mobile element. Yellow arrows indicate open-reading frames that could not be ascribed resistance or mobility but were interspersed between other genes of interest. A full annotation of these regions is not included to highlight areas of greater interest.

We analysed 12 *mcr-10* positive isolates with long-read data available (Fig. 6), three contained the *mcr-10* on the chromosome, nine isolates contained the *mcr-10* gene on plasmids. There was one additional isolate (NK-MR1610) that contained an *mcr-10-*like gene on a 15,560 bp contig; however, it only had 94% identity and 15 amino acid polymorphisms indicating it could not be considered a true *mcr*−10 gene. Furthermore it was not preceded by tyrosine-type recombinase/integrase (GenPept accession WP_032676104.1), conserved in all other *mcr-10* carrying isolates (Fig. 6). The local environment of this tyrosine-type recombinase/integrase and *mcr-10* combination was only found between mis-matched IS elements that ranged in size from 5132 bp to greater than 50,000 bp (Fig. 6A–D). Three of the plasmids with *mcr-10* (NK-1540, NK-MR6054, and NW-MR576) (Fig. 6C, D) and NW-MR132 with *mcr-10* chromosomally located shared homologous regions between 23,881–25,447 bp with an *Enterobacter roggenkampii* strain 120063 plasmid (GenBank CP116250) including an operon of tellurite resistance genes ending at a ISKpn*74* transposase, suggesting a common origin. Analysing the regions surrounding *mcr-10* for all plasmids and chromosomal insertions using Isfinder (https://isfinder.biotoul.fr/) identified flanking transposases demarcating islands from 5903 bp to 65,813 bp containing *mcr-10*. However, no consistent pattern could be discerned as individual IS elements (including IS*15DIV*, IS*Kpn26*, IS*Kpn74* and IS*1B*) were only found in a maximum of *n* = 3/12 isolates and the lack of matching IS elements indicates they would be unable to move further.

### Phylogenetic analysis of *Enterobacter* species

**Fig. 7:**
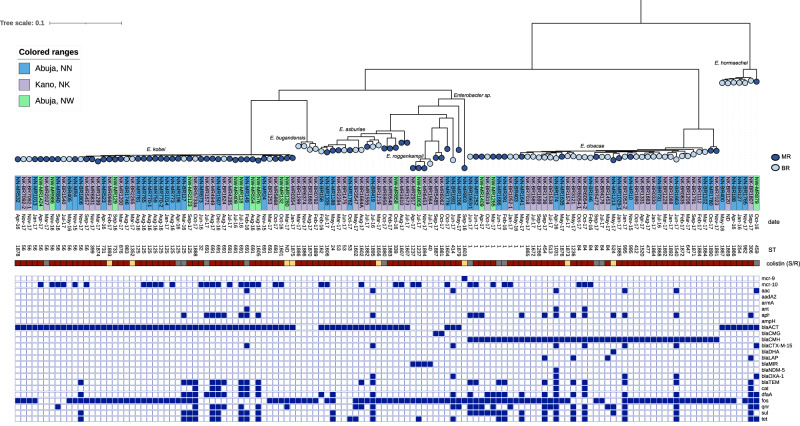
A core genome characterization of 130 *Enterobacter* spp. isolates using Panaroo to generate a core genome alignment and IQtree to build the phylogeny. The colours in the circles indicate the sample type (MR, mother rectal or BR, baby rectal). The colour of the label which represents the isolate identification code (leaf) denotes the hospital location (NN, NW & NK). The date displayed is the month of the rectal swab collection. The sequence type (ST) of the isolate is denoted in the numerical text following the isolate date. The colour strip represents colistin sensitivity/resistance with red denoting resistance, yellow sensitivity, and grey indicates data not available. Followed by a heatmap of resistance genes, beginning with the presence of *mcr* gene, and ARGs were grouped per antibiotic class.

A core genome phylogenetic tree of 130 *Enterobacter* isolates obtained during this study (70 MR and 60 BR isolates) was produced to evaluate species wide diversity amongst the *mcr-*positive and *mcr-*negative colistin resistant bacteria (Fig. 7). Collectively, the most frequent species were *E. kobei* and *E. cloacae* (*n* = 49 and *n* = 44 respectively), which represented 72% of the total dataset, but nine distinct colistin resistant species of *Enterobacter* were present (Fig. 7). There was a large diversity with 74 distinct STs and the most common across both *mcr* positive and negative isolates were ST125, ST56, ST691, ST1 and ST84. A majority of BR *Enterobacter* were *E. cloacae* (Fig. 7) whereas the majority of *E. kobei* were from MR samples. ST125 *E. kobei* (*n* = 10) were recovered from Abuja (both in NN and NW), seven contained *mcr-10* and three were negative however all were resistant to colistin (Fig. 7). SNP analysis (NN-MR773 used as the reference, single contig chromosome 4,382,297 bp) revealed that *n* = 6/7 *mcr-10* positive isolates, collected from the same recruitment site (NN, Abuja) in August 2016 were within 10 pairwise SNPs (each to NN-MR773) suggesting clonal spread or a single acquisition point perhaps within the hospital. The single remaining *mcr-10* positive ST125 *E. kobei* was also collected from NN in April 2016 however was >800 SNPs distant from NN-MR773. Likewise, the *mcr-10* negative ST125 *E. kobei* from NN, were also >700 SNPs distant from *mcr* positive isolates. The single *mcr* negative ST125 *E. kobei* from the second hospital site in Abuja (NW) was over 1800 pairwise SNPs distant from all *E. kobei* isolates collected from NN.

Two *E. cloacae* isolates (NN-MR1780 and NK-MR7023) sit on a separate branch distinct from the remaining (*n* = 42) *E. cloacae*. Most isolates contained *bla*_ACT/MIR_ as these are often intrinsic in multiple *Enterobacter* species, however there were four isolates of *E. bugandensis* that sat on a distinct branch, and a further single isolate (Fig. 7) identified as a partial match to *E. cloacae*, that did not contain any genetic markers conferring resistance to ß-lactams. The unidentified *Enterobacter* species carries *mcr-9.1* and was susceptible to colistin. The remaining (*n* = 7) isolates susceptible to colistin (one carried *mcr-10*) were different species across the phylogeny.

### Conjugation of *Enterobacter* carrying *mcr-9* and *mcr-10*

To determine whether *Enterobacter* species could be acting as a reservoir genus, we performed conjugation experiments on seven distinct bacterial isolates (denoted in Table 2) with a range of plasmid Inc types including IncFIB and IncFII with plasmid sizes between 65,562 bp to 191,776 bp (denoted in Table 2). Two plasmids (pNK-BR1684 and pNN-BR1663r1; Fig. 6) contained relatively complete cassettes of transfer or *tra* genes that had the potential for functionality; while pNN-MR-773 and pNW-MR-576 carried truncated portions of the cassette (*dbs*A with either *traA*/*traL*/*traE* or *traI*/*traD*, respectively) that lack pilus or secretory type IV genes that would make it functional for plasmid transfer^41^. Further analysis of NK-BR1684 identified a 36,594 shared region of homology with the *Enterobacter* spp. Z1 unnamed plasmid 2 (GenBank accession number CP099720) which also contains conjugal transfer proteins *traC*, *traD*, *traF*, *traG*, *traH*, *traI*, *traN*, *traT* and *traU*. However, after multiple attempts of different donor recipient cell density ratios, it was not possible to conjugate any of the plasmids containing an *mcr* gene from *Enterobacter* to *E. coli* J53.

## Discussion

To the best of our knowledge, this is the largest screening of intestinal microbiota for colistin resistance in Nigeria analysing almost 5000 samples. We found an *mcr* carriage of 1%, similar to our recent study which screened the same cohort of mothers and neonates for the carriage of carbapenemase ARG and reported *bla*_NDM_ and *bla*_OXA-48_-like at 1.5% and 2% respectively^28^. Although data on colistin resistance in Africa is scarce, there has been a noticeable increase in the last three years. In 2021, Ugah and Udeani reported high phenotypic resistance to colistin in Nigerian clinical isolates^42^ and suggest a discord between colistin resistant bacterial infections and evidence of colistin antibiotic therapy, suggesting non-therapeutic colistin pressures are creating silent reservoirs. Furthermore, a recent study by Umair et al., 2023 indicates that there are multiple European import sources of colistin into Nigeria, likely for use in agriculture^43^. We detected *mcr-1.1 E. coli* and *K. quasipneumoniae* from a sample collected in November 2015, suggesting colistin resistance has been present in the Nigerian community for years and the microbiota is a likely reservoir. Mothers may be acquiring colistin resistant bacteria from the community, however neonatal acquisition may be from the community, maternal vertical transmission, or the environment. In 2018 Anyanwu et al., screened for colistin resistance in 785 cloacal swab samples from the South of Nigeria and similarly detected *mcr-1.1* in *E. coli* and *K. pneumoniae* isolates further suggesting the spread of *mcr* in Nigeria across sectors^19^.

We found a microbial growth rate of 70% following enrichment in colistin broth (4 mg/mL), similar to previous studies which found broth enrichment steps can maximise *mcr* recovery^44–46^. While the MIC for the *mcr*−9 carrying isolate NN-MR803 was initially found to be below the threshold for colistin resistance (>2 mg/L), we found that a single passage in sub-MIC concentration elevated the subsequent MIC to 8 mg/L (now resistant) and a second sequential sub-MIC exposure raised the MIC to 32 mg/L (Supplementary Fig. 2). These results indicate that exposure to stepwise increases in antimicrobial concentrations in vitro may miss resistance genes that are regulated by either inducible promotors or require alteration to gene repressor mechanisms that are sensitive to gradual increases in antimicrobial concentrations. Therefore, while we did not interrogate the colistin susceptible isolates carrying *mcr*−10, it is possible that pre-MIC incubation with low levels of colistin may have resulted in emergence of resistant phenotypes for these isolates as well. This has been noted by other studies indicating microbiological and genomic surveillance of colistin resistance is essential to capturing all potentially resistant bacterial populations^40,47–50^ Whilst there are many studies utilising real-time PCR as an effective *mcr* screen^51,52^, this is costly compared to end-point PCR. An initial molecular and low-cost screen as implemented in this study can be effective and sustainable to perform in laboratories where resources and access may be restricted. It is not practical to screen all *mcr* genes, however *mcr-1, mcr-3, mcr-8, mcr-9*, and *mcr-10* are often considered the most clinically important^53^.

Throughout the BARNARDS study we have demonstrated that *Enterobacter* is a clinically important genus, and important in the context of AMR. Phylogenetic analysis revealed large intraspecies diversity of colistin resistant *Enterobacter* suggesting a diverse reservoir of colistin resistant bacteria within the microbiota of women and neonates, with *mcr* genes being identified within multiple species. *Enterobacter* is an ESKAPE pathogen, is often antimicrobial resistant and is responsible for many nosocomial infections^54^. *Enterobacter* acquiring ESBL and carbapenemase ARG were amongst the top five non-outbreak GNB causing neonatal sepsis^26^, and top three within the mother/neonatal gut^28^. Furthermore, Liao et al., 2022 reported a high prevalence of colistin resistance and *mcr-9/mcr-10* in *Enterobacter* over a decade in China^55^. More concerning however is a recent Nepalese study by Manandhar et al., 2022, where they describe a fatal neonatal sepsis outbreak caused by *E. kobei* carrying *mcr-10* and the authors hypothesised hospital surface colonisation via the creation of biofilms^56^.

We were not successful in conjugating any plasmids carrying *mcr-10* into *E. coli* J53, similar to other reports and this suggests that *mcr-10* may be, at least for now, a concern largely limited within *Enterobacter* species. If conjugation of *mcr-10* into J53 had been successful, we would have attempted to conjugate *mcr-10* into wildtype strains of *E. coli* and *K. pneumoniae*, as along with *Enterobacter*, these species were the dominant AMR resistant isolates recovered during clinical and carriage BARNARDS samples^26,28^. Whilst the body of current literature primarily links *mcr-10* to *Enterobacter* species, there are reports of plasmid mediated *mcr-10* detected in other species including *Cronobacter*, *Klebsiella*, and *E. coli*^57,58^. In this study, we identified isolates containing *tra* genes which may assist conjugation of the *mcr* containing plasmid. Furthermore, all *Enterobacter* with additional long-read data carried a tyrosine-type recombinase/integrase upstream of the *mcr-10* gene which may, contribute to the mobilisation of *mcr-10* gene as suggested previously^57,58^ given previous evidence of an upstream *xerC* involvement in mediating the carbapenemase resistance gene *bla*_IMI_^59^. There are multiple mechanisms of colistin resistance in *Enterobacter* species aside from the acquisition of *mcr* gene suggesting that there may be undetected reservoirs of colistin resistance in the gut microbiota. Previous studies suggest that chromosomal mutations conferring resistance to colistin (*pmrA/pmrB*, *phoQ*, and *mgrB* genes) may be present in *Enterobacter*^47,60^ and future work to determine chromosomal mutations conferring colistin resistance are needed.

A limitation of this study was a lack of colistin susceptible isolates due to our laboratory approach. Long-read sequences were generated based on initial short-read bioinformatics analysis, therefore it was not possible to characterise the genetic context of all *mcr* genes. We did not collect data on the human immunodeficiency virus (HIV) status of the mother, and this, along with other conditions affecting the immune system may be drivers for AMR carriage. A further limitation was the different sample size of rectal swabs processed per site. All BR samples were processed. MR samples were processed in proportion to the total number of women enrolled into the study (NN *n* = 909/1902, NW *n* = 919/2359, and NK *n* = 2359/7319 samples processed). The prevalence of *mcr* must therefore be interpreted with caution accounting for the variation in the overall sample size per site and all findings are presented descriptively.

In conclusion, *mcr* carriage was higher in urban settings between 1.3–2% in Abuja compared to 0.5% in Kano. The dominant reservoir of colistin resistant species in the microbiota were *Enterobacter* spp., and worryingly, we detected *mcr* positive GNB in neonates less than a week old, including neonates presenting with sepsis. The majority of *mcr* genes were located on plasmids, which may, conditions permitting, disseminate wider in the intestinal microbiota and surrounding environments. The large diversity of the *mcr* genetic context is concerning as there are likely multiple acquisition routes suggesting that ongoing colistin resistance surveillance is vital, particularly in vulnerable population groups such as neonates. Unfortunately, due to data indicating agricultural use of colistin in Sub-Saharan Africa, these findings could likely be extrapolated across Africa and are, regrettably, higher in Asia where there is greater evidence of colistin usage agriculturally and colistin resistant infections.

## Methods

### Study design

A prospective cohort study was conducted through the BARNARDS network consisting of 12 clinical sites in seven countries in Africa (Ethiopia, Nigeria, Rwanda, and South Africa) and South-Asia (Bangladesh, India, and Pakistan), this work focuses exclusively on the three sites in Nigeria. Rectal samples were taken from all mothers upon recruitment and from neonates with clinical diagnosis of sepsis and upon clinical discretion from birth up to 60 days old as described in Carvalho et al., 2022^28^. Samples were maintained at 4 °C until transfer to Cardiff University (CU) under UN3733 regulations.

Clinical variables analysed in this manuscript include admission date, enrolment date, neonatal and maternal (within last three months) antibiotic use, neonate’s date of birth, place of birth (cohort), and neonatal outcome (alive/deceased). Ethical approval was sought from each institution; NK Kano State Hospitals Management Board 8/10/1437AH 13/07/2016, NN Health Research Ethics Committee (HREC), National Hospital, Abuja NHA/EC/017/2015 27/04/2015 and NW Health Research Ethics Committee (HREC), National Hospital, Abuja NHA/EC/017/2015 27/04/2015. Consent was procured in writing in local languages by research nurses providing mothers with study information, consenting for both mother and/or neonatal enrolment. If written consent not obtainable (due to literacy barriers) and oral consent was collected from the mothers by trained researchers. Oral consent was documented by the participant signing/marking the consent form as reported in Carvalho et al., 2022^28^.

### Processing rectal swabs and bacterial isolates

We selected samples proportionally across the sampling time period from November 2015-December 2017. All BR swabs collected during BARNARDS were included for *mcr* screening. MR and BR were cut into 5 mL of Lysogeny Broth (LB) with vancomycin (10 mg/mL) and colistin (4 mg/mL)^44^ and amphotericin B (1 µg/mL) and incubated at 37  °C shaking at 180 rpm for 24 h. Swabs with no growth after 24 h were further incubated to 48 h. 1 mL of broth was transferred to a deep well MIDI 96-well plate and was centrifuged at 1530 × g for 10 min to pellet microbial growth. The supernatant was removed, and the pellet was washed with 0.2 mL 1× PBS to remove excess charcoal. The MIDI 96-well plate was further centrifuged at 1530 ×g for 1 min. The PBS was removed, and the pellet resuspended via pipette mixing in 0.1 mL of molecular grade water. The 96-well plate was centrifuged at 1530 × g for 1 min resulting in the gDNA template for PCRs. PCR conditions for *mcr-1*, *mcr-8*, *mcr-9*, and *mcr-10* are detailed in Supplementary Table 1. Gel electrophoresis was performed with a 1.5% agarose gel for 35 min at 250 v and imaged using a G:BOX Chemi-XX6 GENESys, (Cambridge, UK). 50 μL of the overnight culture of rectal samples with a positive PCR result for any *mcr* variant were streak plated onto a UTI agar plate (Sigma) supplemented with vancomycin (10 mg/ml) and colistin (2 mg/ml). Each phenotypically different colony was sub-cultured, and the PCR was repeated for the gene(s) of interest. All positive bacterial isolates were identified using Microflex LT MALDI-TOF MS (Bruker Daltonik, GmbH, UK) with α-Cyano-4-hydroxycinnamic acid (HCCA) matrix (Sigma Aldrich) and preserved in TS/72 beads (Technical Service Consultants, UK) at −80 °C for gDNA extraction and a final confirmation of the *mcr* gene (for the positive isolates) before whole genome sequencing (WGS). A selection of bacterial isolates (selected across the sampling time and at a ratio of 2:1 negative to positive) that grew in the presence of colistin (4 mg/mL) were also preserved in TS/72 beads (Technical Service Consultants, UK) at −80 °C for gDNA extraction and WGS for comparative analysis and to look for chromosomal genes conferring resistance to colistin.

### Antimicrobial susceptibility testing

Minimum inhibitory concentrations (MICs) were determined for *mcr* positive and *mcr* negative isolates purified from rectal swabs by in-house agar dilution for four antibiotics: colistin, gentamicin, ampicillin, and meropenem. *E. coli* ATCC 25922, *E. coli* NCTC 13846 (*mcr-1* positive) and *Pseudomonas aeruginosa* ATCC 2785 were used for quality control and interpreted according to the EUCAST v12 guidelines^61^. Each bacterial isolate was tested in triplicate per antibiotic concentration to generate three separate data points following validation using appropriate control strains listed above from which a median value was used in Fig. 3a. Additionally, NW-MR803 (*mcr-9* positive) was subjected to a three-day serial passage in a sub-MIC of colistin to determine whether resistance was inducible. MICs were determined according to EUCAST guidelines as above in Mueller-Hinton broth for colistin (0.06–128 mg/L range), comparing the original isolate to 1-day, 2-day and 3-day sub-MIC colistin induced isolates, in parallel and in triplicate. Consistent relative increases in MIC plateaued after 2 days induction (16-fold relative to original isolate).

### Bacterial conjugation

Donor isolates were plated onto colistin agar (2 mg/L). J53 was used as the recipient for all experiments and was cultured onto UTI ChromoSelect agar (Sigma, UK) supplemented with 100 mg/L sodium azide (NaN3). Overnight cultures of donor and recipient isolates were prepared and diluted 1:100 in LB broth and incubated at 170 rpm at 37 °C until in log phase growth (OD 0.4–0.6 at 600 nm). The donor and recipient were mixed at ratios 1:3, 1:5 and 1:1 for each experiment and the mating cultures were incubated at 37 °C for 16–20 h. Transconjugates were selected for by plating 100 mL of suspension onto agar supplemented with NaN3 (200 mg/L) and colistin (2 mg/L). The conjugation frequency was calculated by counting CFU of donor and transconjugates, these were confirmed by PCR. A positive *mcr-1* control plasmid (*E. coli* bacteria) was included in each experiment to calculate the conjugation frequency.

### WGS and bioinformatics analysis

A colony was transferred into 1.8 mL of LB broth with colistin at 2 mg/L and incubated at 37 °C, 180 rpm for 18 h. gDNA was extracted and genomic libraries were prepared exactly as described in Carvalho et al., 2022^28^. The same gDNA was used to generate complimentary long read sequencing data for selected isolates. SPRI beads (Mag-Bind TotalPure, Omega) were used to concentrate and purify at a 1:1 ratio with a final elution volume of 15 µL to achieve an optimal range between 40–60 ng/µL. gDNA was quantified with the dsDNA broad range assay kit using the qubit 4.0 fluorometer (Thermofisher). Genomic libraries were prepared using the Rapid Barcoding Kit (SQK-RBK004; Oxford Nanopore) with an extended incubation of 30 min following addition of RAP and sequenced on a R9.4 flow cell using a MinION (Oxford Nanopore). For all short reads we sequenced to an overall genome coverage of 30X. For all *mcr* isolates, additional long reads were generated to increase the hybrid genome coverage to >50–100X.

Bioinformatics was performed as described in Carvalho et al., 2022^28^. Trimgalore (v0.6.4) was used to remove the Nextera adapter sequences and low-quality bases (--paired --phred33 -q 25 --illumina -e 0.2). Reports were generated using fastqc (v0.11.8) and collated using MultiQC (v1.12). Bacterial species were identified using Blastn nt (v2.7.1) and PathogenWatch (v.15.0.2). Genomes were screened for ARG using ABRicate (v1.0.0) with associated databases NCBI (Database accessed June 2022) and resfinder (Database accessed 2022) (98% identity/coverage cut off). Each genome was screened in the two different databases (NCBI and resfinder; both available within ABRicate) and cross checked for consistency. Previously undetected alleles, and ST profiles were submitted to BIGSbd and PubMLST for assignment. For all *Enterobacter* isolates, a core genome alignment was generated using Panaroo (v1.2.10), and a phylogenetic tree was constructed using IQtree (v2). Phylogenetic trees were mid-rooted, visualised, and annotated using iTOL (v6.0). Guided by ST data and the phylogenetic tree, secondary analysis was performed to determine the isolate relatedness for ST125 *E. kobei*. For this isolate relatedness analysis, Snippy (v4.6.0) was used with default parameters to call pairwise SNPs for ST125 *E. kobei* isolates using NN-MR773 as the reference. Additionally, snippy (v4.6.0) was also used to assess SNPs between complete plasmids (resulting from hybrid assemblies) and Illumina short-reads of corresponding isolates.

FAST5 reads were basecalled using Guppy (v4.5.4) within MinKnow and FASTQ reads were trimmed with Filtlong (v0.2.0) (--min_length 1000 –min_mean_q 10). Unicycler (v0.4.7)^62^ was used to hybrid assemble the short-read and long-read. Genomes were annotated as described above. Where hybrid assemblies produced circularisation of chromosome and/or plasmid contigs this was confirmed using Bandage (v0.9.0) and Geneious (v2022.1.1). Isolates for which Unicycler assemblies contained homopolymer-related open reading frame breaks (NW-MR803, NN-MR49-1 and NN-MR49-2) were re-sequenced on both MiSeq and MinION platforms in August 2023 for confirmatory assemblies. Confirmatory long-reads were basecalled using Guppy (v6.5.7) with an additional trimming argument (--num_extra_bases_trim 20) and short-reads were trimmed using Trim Galore (v0.6.10) using default settings. Confirmatory assemblies were performed using the Dragonflye (v1.1.1) wrapper to perform additional trimming with fastP (v0.23.4) and porechop (v0.2.4) before executing a Flye (v2.9.2) assembly with two rounds of Medaka (v 1.8.0) polishing and one round of Polypolish (v0.5.0) polishing. Plasmid contents of Dragonflye assemblies were further investigated using a specialist plasmid assembler, plassembler (v1.2.0), to confirm Flye assemblies were not missing plasmids. Multiple QC stages were reviewed in MultiQC (v1.12) using default QC collation metrics.

Genomes were downloaded from NCBI for comparative analysis and collections were screened for the presence of *mcr* genes using ABRicate (v1.0.0). Genomes were annotated using Prokka (v1.14.5)^63^, ISfinder^64^ (https://www-is.biotoul.fr/index.php; Database accessed May 2023) and Blastn (nt database) within Geneious. Each identified IS was downloaded into Geneious and used to annotate the genomes. All additional annotations were performed by Geneious by downloading closest homologous GenBank deposited genomes using the integral Blastn tool in Geneious to search NCBI databases. Progressive Mauve^65^ (Geneious plugin) was used to compare plasmid sequences and genetic context images were created from Geneious outputs in Adobe Illustrator (v26.3.1). PLSDB (https://ccb-microbe.cs.uni-saarland.de/plsdb/; Database accessed May 2023) and Blastn (nt database) were used to search for plasmids for comparative analyses. Bioinformatics analysis was performed using a high-performance computing cluster at Cardiff University (ARCCA).

### Statistics & reproducibility

No statistical method was used to predetermine sample size. No data were excluded from the analyses, the experiments were not randomised, and the investigators were not blinded to allocation during experiments and outcome assessment.

### Reporting summary

Further information on research design is available in the Nature Portfolio Reporting Summary linked to this article.

### Supplementary information

Supplementary Information

Description of Additional Supplementary Files

Supplementary Data 1

Supplementary Data 2

Reporting Summary

## Data Availability

All short-read sequences generated were submitted to the European Nucleotide Archive (ENA) and given the project number PRJEB44720 (Supplementary Data 2). All hybrid assembled genomes (from short-read and long-read) were uploaded to NCBI and given the project number PRJNA860154. All raw MIC data is available within the Supplementary Data 1 file. Isolate availability will be considered by the authors under the remit of a mutually accepted material transfer agreement.

## Code availability

All bioinformatics software used has been extensively mentioned in the methods section. All default parameters used unless specifically detailed in the methods section and therefore no bespoke code was generated during this study.
