# Supplementary material for: Characterisation of colistin resistance in Gram-negative microbiota of pregnant women and neonates in Nigeria
Source: Nat Commun. 2024 Mar 14;15:2302. doi: 10.1038/s41467-024-45673-6 (PMC10940312; doi:10.1038/s41467-024-45673-6)
Supplement: Supplementary file 1 — Supplementary Information [file 41467_2024_45673_MOESM1_ESM.pdf]

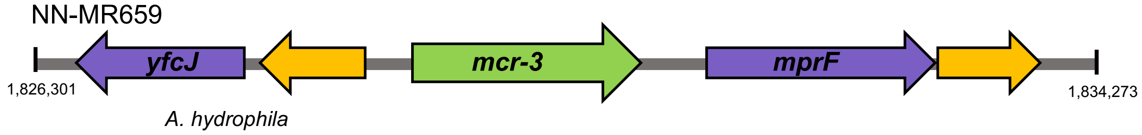

Supplementary Figure 1: Highlights the *mcr-3* A*eromonas hydrophilia* NN-MR659 isolate which is flanked by genes *yfcJ* upstream and *mprF* downstream.

Supplementary Discussion

***mcr-3* like genetic context**

One *A. hydrophila* isolate cultured from an MR swab was positive for a chromosomal *mcr-3*-like gene (NN-MR659, genbank accession: CP124746). From 264 available *A. hydrophila* genomes in NCBI, only n=4 contained *mcr-3* genes (CP028567.2, AP022206.1, VHIW01000011, AP025277, JARESK010000048). Further analysis showed a single amino acid difference (I166L), with a total of 25 SNPs between the *mcr-3* like gene detected in this study to an *A. hydrophila* isolate (Genbank accession number CP027804). The *mcr-3* like gene (1,626 bp) showed a 94.1-94.8 % amino acid identity to proteins found in three *Aeromonas* species: one *A. hydrophila* isolate from human peritoneal fluid (NZ_AOBN01000008.1), one *Aeromonas caviae* isolate from lake water in Malaysia (NZ_JWJP01000016.1) and one *Aeromonas media* isolate of unknown origin (NZ_CDBZ01000089.1), suggesting it might be a common gene in *Aeromonas* species. The *mcr-3* gene for the three *Aeromonas* isolates was between an *eamA* and a diacylglycerol. There were no integrases or insertion sequences around the *mcr-3*.

Aeromonas and *mcr* discussion

Interestingly, we detected an *mcr* positive *A. hydrophila* isolate through our *mcr* PCR screening method and Aeromonas have been linked as the origin species for *mcr-3*[1]. Further sequencing has cast doubt on whether this is a true *mcr* gene, or whether this is an associated phosphoethanolamine gene. There are now several reports on colistin resistant Aeromonas species in the environmental context, particularly the aquatic environment, however Komeda *et al.,* 2022 recently published data where they identified clinical Aeromonas harbouring *mcr* like genes, or genes encoding phosphoethanolamine transferases[2]. Whilst *mcr-3* might not be the primary cause for colistin resistance in Aeromonas, as the insertion of IS elements may have interrupted gene expression as discussed by Xu *et al*[3], the presence of *mcr-3* genes in Aeromonas isolates from both a clinical origin presents a potential reservoir of colistin resistance warranting ongoing surveillance[4].

a)

b)

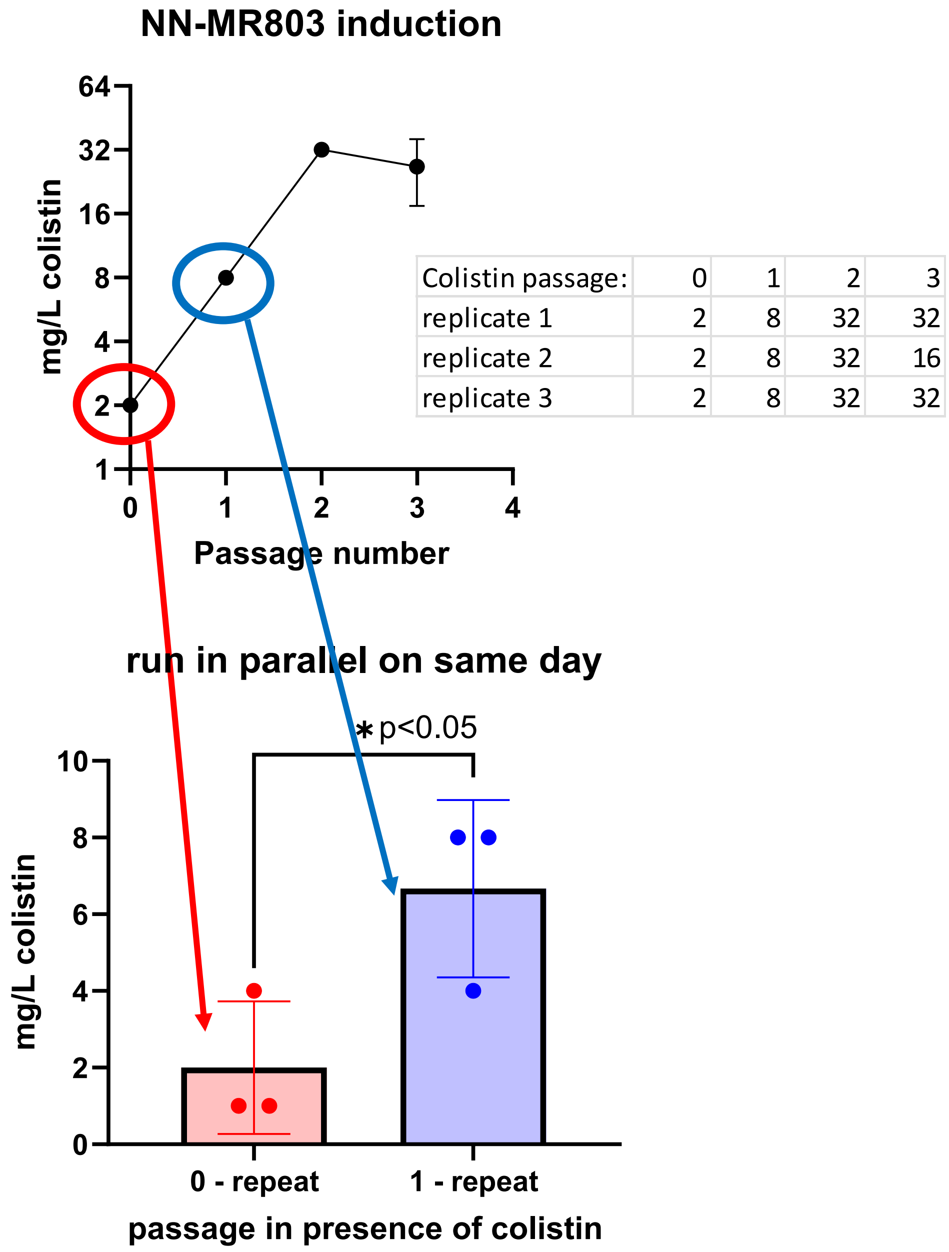

Colistin passage:	0	1	2	3
replicate 1	2	8	32	32
replicate 2	2	8	32	16
replicate 3	2	8	32	32

Supplementary Figure 2: Showing colistin resistance induction for *mcr*-9 positive isolate NN-MR803.

Baseline MIC for colistin was higher (2 mg/L) in broth microdilution than in previous agar dilution screening (0.5 mg/L).

(a) Serial sub-culture and repeat MIC from last growth well showed 4-fold increase after one passage and a further 4-fold increase after two passages in sub-MIC colistin (0.5 mg/L). MICs performed at 24 hour intervals, using growth at 0.5 mg/L well as source of bacteria for each subsequent day. All MICs replicated in triplicate.

b) Colistin MIC for parent NN-MR803 isolate (sub-cultured in absence of colistin), run in parallel on same day as isolate that was sub- cultured with 0.5 mg/L colistin overnight prior to MIC. Student t-test comparison shows statistically significant increase in MIC ($p<0.05$) when MICs performed in parallel in triplicate.

Supplementary table of primers and conditions

Target	Sequence (5'-3')	Conditions	Amplicon size (bp)	Reference
mcr-1	F- AGTCCGTTTGTTCTTGTGGC<br>R- AGATCCTTGGTCTCGGCTTG	94ºC— 5 minutes<br>{94ºC 45 seconds<br>30X — {59ºC 30 seconds<br>{72ºC 30 seconds<br>72ºC— 10 minutes<br>4ºC — ∞	320	(Rebelo *et al*, 2018)[5]
mcr-8	F-CGTACAGGTGTTGAGGTGCT<br>R-GCATCCCGGAATAACGTTGC	95ºC— 5 minutes<br>{95ºC 30 seconds<br>35X — {59ºC 30 seconds<br>{72ºC 30 seconds<br>72ºC— 10 minutes<br>4ºC — ∞	403	This study
mcr-9	F-TACCGGTATCCTTCCTGCCA<br>R-ACAACCGCCATCGTTCTCTT	95ºC— 5 minutes<br>{95ºC 30 seconds<br>35X— {59ºC 30 seconds<br>{72ºC 30 seconds<br>72ºC— 10 minutes<br>4ºC — ∞	595	This study
mcr-10	F-CTCGCTTCGCTGATCCTGAT<br>R-CGCTGGTAATAGGTCGGTCC	95ºC— 5 minutes<br>{95ºC 30 seconds<br>35X— {59ºC 30 seconds<br>{72ºC 30 seconds<br>72ºC—10 minutes<br>4ºC — ∞	689	This study

Supplementary References

1. Yin, W. *et al*. Novel Plasmid-Mediated Colistin Resistance Gene *mcr-3* in *Escherichia coli*. mBio 8(3), e00543-1 (2017).
2. Komeda, T. *et al*. Emergence of a highly colistin-resistant *Aeromonas jandaei* clinical isolate harbouring four genes encoding phosphoethanolamine transferases in Nepal. Int. J.Antimicrob. Agents 59(4), 106544 (2022).
3. Xu, L. *et al*. The variants of polymyxin susceptibility in different species of genus Aeromonas. *Front. Microbiol*. 13, 1030564 (2022).
4. Shen Y., *et al*. Prevalence and Genetic Analysis of *mcr-3*-Positive Aeromonas Species from Humans, Retail Meat, and Environmental Water Samples. *Antimicrob Agents Chemother*. 27;62(9), e00404-18 (2018).
5. Rebelo, A.R. *et al*.. Multiplex PCR for detection of plasmid-mediated colistin resistance determinants, *mcr-1*, *mcr-2*, *mcr-3*, *mcr-4* and *mcr-5* for surveillance purposes. *Euro surveillance* 23, 17. (2018).
