# Supplementary material for: Characterisation of colistin resistance in Gram-negative microbiota of pregnant women and neonates in Nigeria
Source: Nat Commun. 2024 Mar 14;15:2302. doi: 10.1038/s41467-024-45673-6 (PMC10940312; doi:10.1038/s41467-024-45673-6)
Supplement: Supplementary file 2 — Description of Additional Supplementary Files [file 41467_2024_45673_MOESM2_ESM.docx]

Description of Additional Supplementary Files

**Supplementary Data 1**

Showing the raw data of the MIC experiments carried out in this work.

**Supplementary Data 2**

Short-read sequences generated submitted to the European Nucleotide Archive (ENA) project number PRJEB44720.
