# Supplementary material for: Characterisation of colistin resistance in Gram-negative microbiota of pregnant women and neonates in Nigeria
Source: Nat Commun. 2024 Mar 14;15:2302. doi: 10.1038/s41467-024-45673-6 (PMC10940312; doi:10.1038/s41467-024-45673-6)
Supplement: Supplementary file 5 — Reporting Summary [file 41467_2024_45673_MOESM5_ESM.pdf]

nature portfolio

Corresponding author(s): Edward AR Portal & Kirsty Sands

Last updated by author(s): 2024/01/22

Reporting Summary

Nature Portfolio wishes to improve the reproducibility of the work that we publish. This form provides structure for consistency and transparency in reporting. For further information on Nature Portfolio policies, see our Editorial Policies and the Editorial Policy Checklist.

Statistics

For all statistical analyses, confirm that the following items are present in the figure legend, table legend, main text, or Methods section.

n/a	Confirmed	
☐	☒	The exact sample size (*n*) for each experimental group/condition, given as a discrete number and unit of measurement
☐	☒	A statement on whether measurements were taken from distinct samples or whether the same sample was measured repeatedly
☒	☐	The statistical test(s) used AND whether they are one- or two-sided<br>*Only common tests should be described solely by name; describe more complex techniques in the Methods section.*
☒	☐	A description of all covariates tested
☒	☐	A description of any assumptions or corrections, such as tests of normality and adjustment for multiple comparisons
☒	☐	A full description of the statistical parameters including central tendency (e.g. means) or other basic estimates (e.g. regression coefficient) AND variation (e.g. standard deviation) or associated estimates of uncertainty (e.g. confidence intervals)
☒	☐	For null hypothesis testing, the test statistic (e.g. *F*, *t*, *r*) with confidence intervals, effect sizes, degrees of freedom and *P* value noted<br>*Give P values as exact values whenever suitable.*
☒	☐	For Bayesian analysis, information on the choice of priors and Markov chain Monte Carlo settings
☒	☐	For hierarchical and complex designs, identification of the appropriate level for tests and full reporting of outcomes
☒	☐	Estimates of effect sizes (e.g. Cohen's *d*, Pearson's *r*), indicating how they were calculated

Our web collection on statistics for biologists contains articles on many of the points above.

Software and code

Policy information about availability of computer code

Data collection	No software was used in the data collection. At the low middle income countries, research nurses completed questionnaires with the women approaching labor. These questionnaires were either transcribed onto paper, due to availability of resources/infrastructure, i.e. Internet access, and later uploaded into Bristol Online survey (BOS) or directly entered into BOS using a tablet device provided by the project.
Data analysis	Trimgalore (v0.6.4)<br>fastqc (v0.11.8)<br>MultiQC (v1.12)<br>Shovill (v0.9.0)<br>quast (v.5.2)<br>Blast nt (https://blast.ncbi.nlm.nih.gov/Blast.cgi) (v2.7.1)<br>PathogenWatch (v.15.0.2; https://pathogen.watch)<br>Snippy (v4.6.0)<br>ABRicate (v1.0.0)<br>BIGSbd (v1.25.1)<br>SeqSero (v1.0)<br>Guppy (v4.5.4)<br>Guppy (v6.5.7)<br>Bandage (v0.9.0)<br>Prokka (v1.14.5)

IQtree (v2.0)
Panaroo (V1.2.10)
iTOL (v6.0)
networkD3 package, Rv3.6.2
Geneious prime (2022.1.1)
fastP (v0.23.24)
porchop (v0.2.4)
dragonfly (v1.1.1)
Flye (v2.9.2)
Medaka (v1.8.0)
plassembeler (v1.2.0)
Polypolish (v0.5.0)

For manuscripts utilizing custom algorithms or software that are central to the research but not yet described in published literature, software must be made available to editors and reviewers. We strongly encourage code deposition in a community repository (e.g. GitHub). See the Nature Portfolio guidelines for submitting code & software for further information.

Data

Policy information about availability of data

All manuscripts must include a data availability statement. This statement should provide the following information, where applicable:

- Accession codes, unique identifiers, or web links for publicly available datasets
- A description of any restrictions on data availability
- For clinical datasets or third party data, please ensure that the statement adheres to our policy

All short-read sequences generated were submitted to the European Nucleotide Archive (ENA) and given the project number PRJEB44720 (Supplementary Table 3). All hybrid assembled genomes (from short-read and long-read) were uploaded to NCBI and given the project number PRJNA860154. All raw MIC data is available within the supplementary information file. Isolate availability will be considered by the authors under the remit of a mutually accepted material transfer agreement.

Research involving human participants, their data, or biological material

Policy information about studies with human participants or human data. See also policy information about sex, gender (identity/presentation), and sexual orientation and race, ethnicity and racism.

Reporting on sex and gender	information on sexual identify was not collected all mothers were recruited without bias. gender of the neonate was recorded.
Reporting on race, ethnicity, or other socially relevant groupings	this data has not been collected
Population characteristics	information on age and other relevant factors were collected in this study. No bias was applied to participant at any stage of the study.
Recruitment	From November 2015 to November 2017, women in labour (preferably) or immediately post partum were recruited prospectively following their consent and their neonate(s) followed up for the first 60d of life or until study withdrawal/neonatal death. as reported in as described in Carvalho et al., 2022 URL:https://www.nature.com/articles/s41564-022-01184-y#Sec10
Ethics oversight	local ethics was obtained as per the BANARDS study<br><br>NK Kano State Hospitals Management Board 8/10/1437AH 13/07/2016<br>NN Health Research Ethics Committee (HREC), National Hospital, Abuja NHA/EC/017/2015 27/04/2015<br>NW Health Research Ethics Committee (HREC), National Hospital, Abuja NHA/EC/017/2015 27/04/2015<br><br>Consent was procured in writing in local languages by research nurses providing mothers with study information, consenting for both mother and/or neonatal enrolment. If written consent not obtainable (due to literacy barriers) and oral consent was collected from the mothers by trained researchers. Oral consent was documented by the participant signing/marking the consent form as reported in Carvalho et al., 2022

Note that full information on the approval of the study protocol must also be provided in the manuscript.

Field-specific reporting

Please select the one below that is the best fit for your research. If you are not sure, read the appropriate sections before making your selection.

☒ Life sciences ☐ Behavioural & social sciences ☐ Ecological, evolutionary & environmental sciences

For a reference copy of the document with all sections, see nature.com/documents/nr-reporting-summary-flat.pdf

Life sciences study design

All studies must disclose on these points even when the disclosure is negative.

Sample size	4,907 rectal swabs (n=3,944 Mother Rectal [MR] and n=963 Baby Rectal [BR]) were processed from hospitals in Kano (NK) and Abuja (NN or NW) (NK-MR n=2,140, NK-BR n=724, NN-MR n=909, NN-BR n=215, NW-MR n=895, NW-BR n=24)<br><br>The sample size was determined by the amount of enrollment. All samples available to test were included, as such no sample size calculations were performed.
Data exclusions	no
Replication	all MICs were performed in triplicate with figures made of the median of the three tests. All replicates were sucessful.
Randomization	as all samples were processed no randomization of samples was possible
Blinding	no blinding was performed in this study, it would not have been feasible with the resources available for this sub-study.

Reporting for specific materials, systems and methods

We require information from authors about some types of materials, experimental systems and methods used in many studies. Here, indicate whether each material, system or method listed is relevant to your study. If you are not sure if a list item applies to your research, read the appropriate section before selecting a response.

Materials & experimental systems

n/a	Involved in the study	
☒	☐	Antibodies
☒	☐	Eukaryotic cell lines
☒	☐	Palaeontology and archaeology
☒	☐	Animals and other organisms
☒	☐	Clinical data
☒	☐	Dual use research of concern
☒	☐	Plants

Methods

n/a	Involved in the study	
☒	☐	ChIP-seq
☒	☐	Flow cytometry
☒	☐	MRI-based neuroimaging
